# Soil bacterial community structure remains stable over a 5-year chronosequence of insect-induced tree mortality

**DOI:** 10.3389/fmicb.2014.00681

**Published:** 2014-12-16

**Authors:** Scott Ferrenberg, Joseph E. Knelman, Jennifer M. Jones, Stower C. Beals, William D. Bowman, Diana R. Nemergut

**Affiliations:** ^1^Department of Ecology and Evolutionary Biology, University of ColoradoBoulder, CO, USA; ^2^Institute of Arctic and Alpine Research, University of ColoradoBoulder, CO, USA; ^3^Department of Plant Biology, University of IllinoisUrbana, IL, USA; ^4^Department of Biology, Duke UniversityDurham, NC, USA

**Keywords:** 16S rRNA gene pyrosequencing, biogeochemistry, community assembly, disturbance ecology, resistance, soil microbial community, soil processes

## Abstract

Extensive tree mortality from insect epidemics has raised concern over possible effects on soil biogeochemical processes. Yet despite the importance of microbes in nutrient cycling, how soil bacterial communities respond to insect-induced tree mortality is largely unknown. We examined soil bacterial community structure (via 16S rRNA gene pyrosequencing) and community assembly processes (via null deviation analysis) along a 5-year chronosequence (substituting space for time) of bark beetle-induced tree mortality in the southern Rocky Mountains, USA. We also measured microbial biomass and soil chemistry, and used *in situ* experiments to assess inorganic nitrogen mineralization rates. We found that bacterial community structure and assembly—which was strongly influenced by stochastic processes—were largely unaffected by tree mortality despite increased soil ammonium (${NH}_{4}^{+}$) pools and reductions in soil nitrate (${NO}_{3}^{-}$) pools and net nitrogen mineralization rates after tree mortality. Linear models suggested that microbial biomass and bacterial phylogenetic diversity are significantly correlated with nitrogen mineralization rates of this forested ecosystem. However, given the overall resistance of the bacterial community to disturbance from tree mortality, soil nitrogen processes likely remained relatively stable following tree mortality when considered at larger spatial and longer temporal scales—a supposition supported by the majority of available studies regarding biogeochemical effects of bark beetle infestations in this region. Our results suggest that soil bacterial community resistance to disturbance helps to explain the relatively weak effects of insect-induced tree mortality on soil N and C pools reported across the Rocky Mountains, USA.

## INTRODUCTION

Bark beetles (Curculionidae: Scolytinae) have killed billions of coniferous trees across North America and Europe in recent epidemics (Meddens et al., 2012; Kärvemo et al., 2014; Latifi et al., 2014). Extensive tree mortality following various types of forest disturbance has been linked to long-lasting changes in terrestrial biogeochemical cycles (Turner, 2010). Bark beetle-induced tree mortality can affect soil properties in a number of ways, with several of the more commonly reported pathways including: (1) the addition of large quantities of nitrogen (N) to the forest floor in dropping needles (Morehouse et al., 2008; Griffin et al., 2011); (2) rapid cessation of root exudates leading to decreased concentration of carbon (C) substrates in soils under dead and dying trees (Xiong et al., 2011); and (3) increased soil moisture and inorganic N concentration as trees cease transpiration and N uptake, respectively (Morehouse et al., 2008; Griffin et al., 2011; Xiong et al., 2011). These biogeochemical changes can enhance mineralization rates, increasing the potential for losses of soil C and N from the system (Turner, 2010; Hicke et al., 2012; Moore et al., 2013; Campbell et al., 2014). Thus, concern over possible impacts of bark beetle-induced tree mortality on terrestrial C and N pools has motivated a number of recent biogeochemical models and studies (e.g., Kurz et al., 2008; Morehouse et al., 2008; Xiong et al., 2011; Edburg et al., 2012; Griffin and Turner, 2012; Moore et al., 2013; Rhoades et al., 2013). Yet, despite the primary roles of microbes in biogeochemical processes, how soil microbial community structure is affected by extensive insect-induced tree mortality remains poorly understood (Štursová et al., 2014).

Microbial community structure can have important influences on ecosystem processes (Monson et al., 2006; Reed and Martiny, 2007; Van der Heijden et al., 2008; Strickland et al., 2009), with shifts in community structure potentially altering ecosystem functioning (Schimel and Gulledge, 1998; Fraterrigo et al., 2006; Schimel and Schaeffer, 2012; Philippot et al., 2013). Meta-analyses of disturbance effects on microbial communities have found they are sensitive to a range of disturbance types, although generalizations may be difficult to make because of publication bias against negative results (Allison and Martiny, 2008; Griffiths and Philippot, 2012; Shade et al., 2012). Also, a shift in both the structure and function of soil fungal communities due to tree mortality from the European spruce bark beetle, *Ips typographus*, has been reported (Štursová et al., 2014). Taken collectively, these results suggest that changes in the structure of soil bacterial communities are a likely outcome of extensive tree mortality during bark beetle epidemics across the USA.

Given reported changes in soil chemistry following bark beetle infestations (Morehouse et al., 2008; Clow et al., 2011; Xiong et al., 2011; Griffin and Turner, 2012; Keville et al., 2013), bark beetle-induced tree mortality might alter the structure of soil bacterial communities via environmental filtering. Alternatively, stronger relative influences of stochastic processes in the assembly of soil bacterial communities have been noted following disturbance (Ferrenberg et al., 2013). An increased influence of stochastic processes could decouple microbial community responses from documented changes in soil chemistry while still leading to shifts in community structure via dispersal, ecological drift or historical contingencies (Nemergut et al., 2013). To understand how tree mortality during bark beetle epidemics affects soil bacterial communities in relation to soil properties and N cycling, we examined bacterial community structure and community assembly processes along a 5-year chronosequence of infestation by the mountain pine beetle (*Dendroctonus ponderosae*). The chronosequence included living control trees (year zero) and trees killed by mountain pine beetles one to 4 years prior to our study (years one–four of the chronosequence). In addition to characterizing bacterial communities, we also measured soil chemical properties of all samples, and completed experimental assessments of N mineralization via *in situ* incubations with soils from a subset of samples in each year of the chronosequence. We tested three primary hypotheses: (1) tree mortality caused a shift in bacterial community structure; (2) the relative influence of stochastic vs. deterministic processes on bacterial community assembly changed over time following tree mortality; and (3) tree mortality led to increased soil N concentrations and N mineralization rates. Finally, we used statistical models to examine the relationships between edaphic factors, soil bacterial community structure and soil processes across the temporal disturbance gradient presented by the chronosequence.

## MATERIALS AND METHODS

### STUDY SITE AND SOILS COLLECTION

Our study was performed on soils from a mature pine forest at the University of Colorado’s Mountain Research Station, 2900 m above sea level and approximately 11 km east of the Continental Divide in CO, USA (40°N; 105°W). The climate and soils of the area were described by Xiong et al. (2011), Mitton and Ferrenberg (2012), and Duhl et al. (2013), and factors underlying bark beetle-induced tree mortality in the site were described by Ferrenberg and Mitton (2014) and Ferrenberg et al. (2014). Work by Monson et al. (2006) and Weintraub et al. (2007) found soil microbial activity in nearby field sites to be greatest under snowpack during spring months. Thus we completed soil sampling in March of 2011, prior to snow melt. Previous work in nearby conifer forests does not indicate the presence of strong, small-scale spatial structuring of soil bacterial communities suggesting minor influences of community autocorrelation within this system (e.g., Ferrenberg et al., 2013). Nevertheless, we selected trees that were scattered across a 2.5 hectare site with an attempt to maximize the distance among trees. All sampled trees were separated by a minimum of 3.5 m and a maximum of 150 m.

**FIGURE 1:**
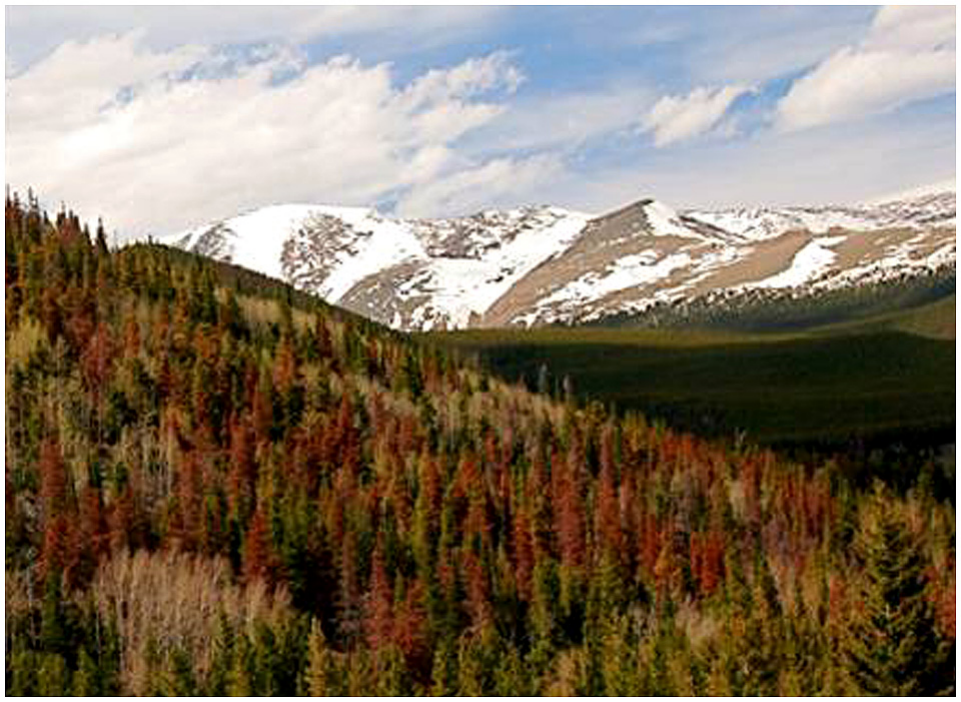
**A recent epidemic of the mountain pine beetle (*Dendroctonus ponderosae*) has led to the deaths of billions of pine trees across western North America.** Rapid warming has allowed the beetle to expand its elevational and latitudinal ranges. This photo shows trees that were attacked 100s of meters above the beetles’ historical elevational range limits in CO, USA. The trees with red needles were attacked during the previous growing season; recently attacked trees have not yet begun to fade (photo by Jeffry B. Mitton).

All trees used in our study had uniquely numbered tags linking them to a forest demography study (see Ferrenberg et al., 2014) that established dates of bark beetle attack and subsequent tree mortality. Tree mortality from bark beetles occurs within a few weeks of mass attack and can be determined from notable changes in needle color (green fades to yellow and red), but does not cause substantial needle drop until the following year (**Figure 1**). We used trees from five temporal categories of bark beetle-induced mortality, which were considered as years 0, 1, 2, 3, and 4 of our 5 year chronosequence. Year 0 corresponded to samples collected from under living trees that were never attacked by bark beetles (we considered these samples as controls), while years 1 through 4 represented samples collected from under trees killed by bark beetles 1–4 years prior to sampling. Over a two day period, we collected a total of 50 soil samples (*N* = 50) which were equally partitioned (10 samples in each) across the 5 years of the chronosequence. Each soil sample was a composite of three, 130.5 cm^3^ cores from the top 5 cm of mineral soil (with all litter and visible organic materials removed) collected roughly 1 m from the base of mature limber pines (*Pinus flexilis*, see Ferrenberg and Mitton (2014) for range and life history descriptions). Following field extraction, all samples were transported on ice, sieved through 2 mm mesh, and a 10 g portion was stored at -80°C for DNA extraction and the remainder at 4°C for biogeochemical analyses. Soils were resampled in June–July 2011 under 25 of the original 50 trees, with five samples from each year of the mortality chronosequence (*N* = 25) used for *in situ* experiments of N mineralization rates and nitrate pools as described below.

### DNA EXTRACTION, PYROSEQUENCING, AND SEQUENCE ANALYSIS

Deoxyribonucleic acid was isolated using the MO BIO Power Soil DNA Extraction kit (MO BIO Laboratories, Carlsbad, CA, USA), and a fragment of the 16S rRNA gene encoding the V1–V2 region was amplified using the primers and PCR steps and program described in Nemergut et al. (2010), Knelman et al. (2012), and Ferrenberg et al. (2013). Three replicate PCR products were quantified, pooled and cleaned using MO BIO UltraClean-htp PCR Clean-up kits and 16S rRNA gene amplicons were sent to the Environmental Genomics Core Facility (Engencore) at University of South Carolina for 454 Life Sciences GS FLX Titanium pyrosequencing.

Pyrosequencing data were screened with the QIIME (Quantitative Insights Into Microbial Ecology) toolkit (Caporaso et al., 2010) with quality score >25, sequence length >200, and <400, maximum homopolymer of 6, 0 maximum ambiguous bases, and 0 mismatched bases in the primer. OTUs (Operational Taxonomic Units) were denoised using Denoiser (Reeder and Knight, 2010) and were picked at the 97% identity level using UPARSE (OTU clustering pipeline; Edgar, 2013). The taxonomic identity of OTUs was assigned using RDP Classifier 2.2 (Wang et al., 2007) with the Greengenes core reference set (DeSantis et al., 2006) in QIIME. Samples were rarefied by randomly subsampling OTUs in QIIME so that each library contained 1100 sequences (the fewest found in a single sample). Quality sequence data were not obtained from one sample in year four of the mortality chronosequence which was excluded from all community analyses. QIIME was also used to generate a UniFrac distance matrix (Lozupone and Knight, 2005) and measures of phylogenetic diversity (PD; Faith, 1992). Sequences and mapping data are available from figshare (Knelman, 2014a,b).

### SOIL ANALYSES

Soil moisture, pH, total %C, and %N, C:N ratio, ${NH}_{4}^{+}$, dissolved organic carbon (DOC), and microbial biomass were quantified for all 50 samples. Soil moisture was determined with the gravimetric method after drying soils at 60°C for 48 h. Soil pH was measured from a 1:5 ratio of soil to distilled and de-ionized H_2_O, and total C and N determined using combustion as described by Knelman et al. (2012). Measures of ${NH}_{4}^{+}$, DOC, and microbial biomass were determined via extractions from 10 g of soil with 0.5 M K_2_SO_4_ as described in Ferrenberg et al. (2013). ${NH}_{4}^{+}$ concentrations were determined using the sodium salicylate method and absorbance at 650 nm on a microplate reader (Mulvaney, 1996). DOC was determined using a TIC/TOC analyzer, with DOC = EC/kEC where EC = extractable C from soil and kEC = extractable C from microbial biomass which was estimated at 0.45 as in Beck et al. (1997). Microbial biomass C was calculated using the chloroform fumigation method (Brookes et al., 1985; Beck et al., 1997) with the procedures described in Ferrenberg et al. (2013). Soil chemistry data are available from figshare (Knelman, 2014b).

To evaluate the influence of tree death on N cycling, net N mineralization rates and concentrations of ${NO}_{3}^{-}$ in ion-exchange resin bags were measured in soils in June–July 2011. Nitrogen mineralization rates were assessed via *in situ* buried-bag incubation experiments. Soil cores (3.5 cm diameter × 10 cm depth) were vertically split and sealed in plastic bags with one half returned to the mineral soil layer and covered with loose soil for 35 days, and the other half used for laboratory analysis to determine starting inorganic N concentrations using the procedures described in Bowman et al. (2006). Inorganic N leaching below the understory plant root zone of each sampled plot (*N* = 25) was measured using two ion-exchange resin bags (Binkley and Vitousek, 1989) inserted at a depth of 15 cm under undisturbed soils and left in place for 41 days prior to removal as described in Bowman et al. (2006). Bags were made of fine mesh nylon surrounded by a plastic cylinder to maintain their structure (4.9 cm^2^ × 2.5 cm tall) and contained mixed-bed ion exchange resins (J. T. Baker, IONAC NM-60 H^+^/OH^-^; Phillipsburg, NJ, USA). ${NH}_{4}^{+}$-N and ${NO}_{3}^{-}$-N from the buried incubation and resin bags was extracted using 2 mol/L KCl and analyzed using a Lachat QuikChem 8000 Spectophotometric Flow Injection Analyzer and a Dionex DX 500 System IonPac AS11 Ion Chromatograph (Sunnyvale, CA, USA), respectively.

### DATA ANALYSES

We compared community structure across years of the tree mortality chronosequence using multi-response permutation procedures (MRPP, a non-parametric method of comparing groups similar to PERMANOVA) and used non-metric multidimensional scaling (NMDS) to visualize the comparisons of community structure. Both MRPP and NMDS were completed on Bray–Curtis distance matrices in PC-ORD (McCune and Mefford, 2011). After verifying that our data met test assumptions of normality via Shapiro–Wilk tests, we compared measures of α-diversity (OTU richness, Pielou’s evenness, and PD) using one-way ANOVA followed by Tukey’s HSD means comparisons. To avoid violating assumptions of sample independence, we compared pairwise community β-diversity (Bray–Curtis dissimilarity) and pairwise UniFrac values using PERMANOVA followed by the permutation method of ‘betadisper’ in the vegan package for the R platform (R Development Core Team, 2011; Oksanen et al., 2013). Comparisons of soil chemistry from bacterial soil samples and from nitrogen resin bags and mineralization experiments were also performed via one-way ANOVA followed by Tukey’s HSD means comparisons (Kruskal–Wallis test followed by Steel-Dwass means comparisons when test assumptions were not met).

We examined possible links between soil bacterial community structure and net N mineralization from incubation experiments (which were completed *in situ* using soils under five of the 10 trees in each year of our chronosequence, *N* = 25) via multiple regression. We considered N mineralization values from all 25 samples as the dependent variable, and used stepwise fitting procedures to determine a best fit model using microbial biomass, bacterial OTU Shannon diversity (H′), bacterial PD, and soil environmental measures (soil moisture, soil temperature, pH, %C, %N, C:N ratio, DOC, ${NH}_{4}^{+}$) as possible independent variables. The best fit model was selected via comparisons of Bayesian information criterion (BIC) values, with the lowest BIC score used to find the model that explained the most variation in N mineralization with the smallest number of retained factors (environmental and/or microbial). Independent variables retained in the best fit model were examined for collinearity via linear regressions, which revealed no significant relationships.

To assess possible correlations between individual environmental factors (%C, %N, C:N, ${NH}_{4}^{+}$, DOC, pH, and soil moisture) and the soil bacterial community, we used Mantel tests to measure associations of the OTUs (determined at 97% similarity) to each of the seven soil environmental variables (i.e., OTUs of each chronosequence year compared to each variable in an individual Mantel test). Mantel tests were completed using 5000 runs with Bray–Curtis distance matrices for bacterial communities and a Euclidean distance matrix for each environmental factor, with a Bonferonni sequential correction used to control for false discovery rate. Additionally, we used the null deviation approach of Chase and Myers (2011) to examine the assembly processes structuring bacterial communities. The null deviation method randomly assembles communities from the regional species pool (all OTUs found among samples) to assess how greatly the observed β-diversity patterns, based on presence-absence data of observed taxa, deviate from stochastic assembly. This approach disentangles the dissimilarity in community composition across samples from variation linked to changes in α- (local) and γ- (regional) diversity (Chase and Myers, 2011). Controlling for changes in local diversity allows us to assess whether changes in β-diversity result from the relative influences of stochastic or deterministic processes. As in Ferrenberg et al. (2013), we calculated null deviation as the relative difference of the observed β diversity from the null-model β-diversity, (β_obs_-β_null_)/ β_null_, with β-diversity measured as Sørenson-Czekanowski binary dissimilarity. Under the null model, expected β-diversity for each sample was calculated from 10000 stochastically assembled communities, and gamma diversity was calculated from the OTU (species) pools within all years of the tree mortality chronosequence. Because the community matrix used in the null devation model is in the form of presence-absence data, taxa with very low abundances (<0.5% of sequences per community) were removed before analyses to avoid the over-weighting of rare taxa (Ofiţeru et al., 2010).

## RESULTS

### BACTERIAL COMMUNITY STRUCTURE AND DIVERSITY

Following rarefaction to an equal sequencing depth of 1100, we found 3126 unique OTUs across all samples of our chronosequence (**Table 1**). There were an average of 1825 OTUs in bacterial communities of each year of the chronosequence, and 736 OTUs shared among all years. We found 124, 139, 160, 178, and 158 unique OTUs across years zero to four of the chronosequence, respectively.

**Table 1:** Soil microbial biomass and measures of bacterial gamma (γ), alpha (α), and beta (β) diversity from a 5-year chronosequence of bark beetle-induced tree mortality.

Years since tree mortality	Biomass (mg/g soil)	γ-diversity	PD (α)	Shannon H’ (α)	Bray–Curtis dis. (β)	UniFrac (β)
0	0.23 (±0.02)	1763	46.3 (±1.07)	8.7 (±0.10)	0.67 (±0.01)	0.23 (±0.010)
1	0.24 (±0.04)	1808	47.3 (±1.40)	8.7 (±0.12)	0.68 (±0.01)	0.24 (±0.005)
2	0.30 (±0.04)	1857	47.7 (±1.43)	8.7 (±0.15)	0.69 (±0.01)	0.24 (±0.014)
3	0.23 (±0.02)	1923	48.1 (±0.95)	8.9 (±0.10)	0.68 (±0.01)	0.22 (±0.012)
4	0.25 (±0.02)	1773	50.7 (±0.56)	8.9 (±0.10)	0.69 (±0.01)	0.24 (±0.017)

*P*-value	>0.10	–	0.084	>0.10	>0.10	>0.10	

*Year 0 = unaffected control trees. Microbial biomass was measured as microbial C from fumigation experiments, γ-diversity indicates the total number of bacterial OTUs found among all soil samples form a given chronosequence year. Alpha and beta diversity measures are calculated as the mean values from all samples in a given chronosequence year: PD indicates the bacterial phylogenetic diversity, H′ shows the Shannon-Weiner diversity index, Bray–Curtis dissimilarity is the pairwise Bray–Curtis dissimilarity index, UniFrac values are weighted by species abundances. Values shown in the table are untransformed means ( ±1 SE).*

**FIGURE 2:**
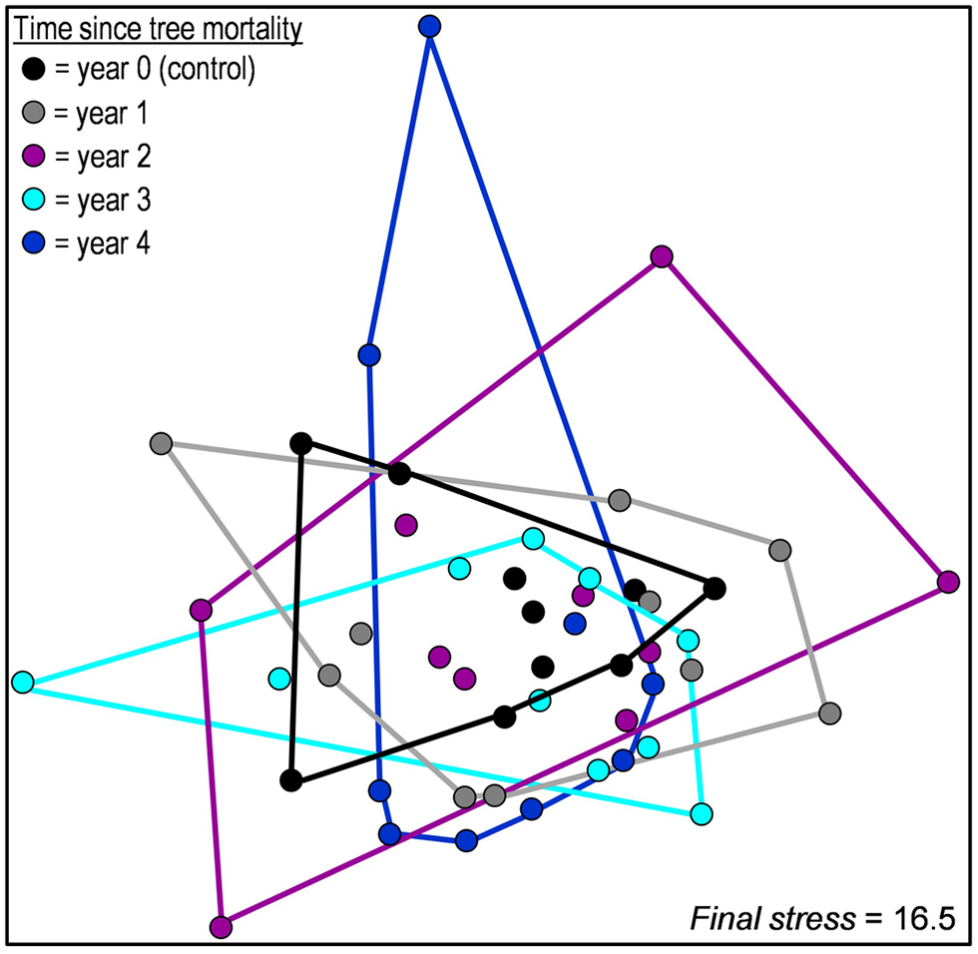
**Non-metric multidimensional scaling ordination based on Bray–Curtis distances comparing the composition of bacterial communities from soils located along a 5-year chronosequence of tree mortality from bark beetle infestations.** Chronosequence year zero is composed of soils located near the trunks of non-attacked, living trees. Years one through four of the chronosequence are composed of trees attacked and killed by bark beetles 1 to 4 years prior to soil sampling.

**FIGURE 3:**
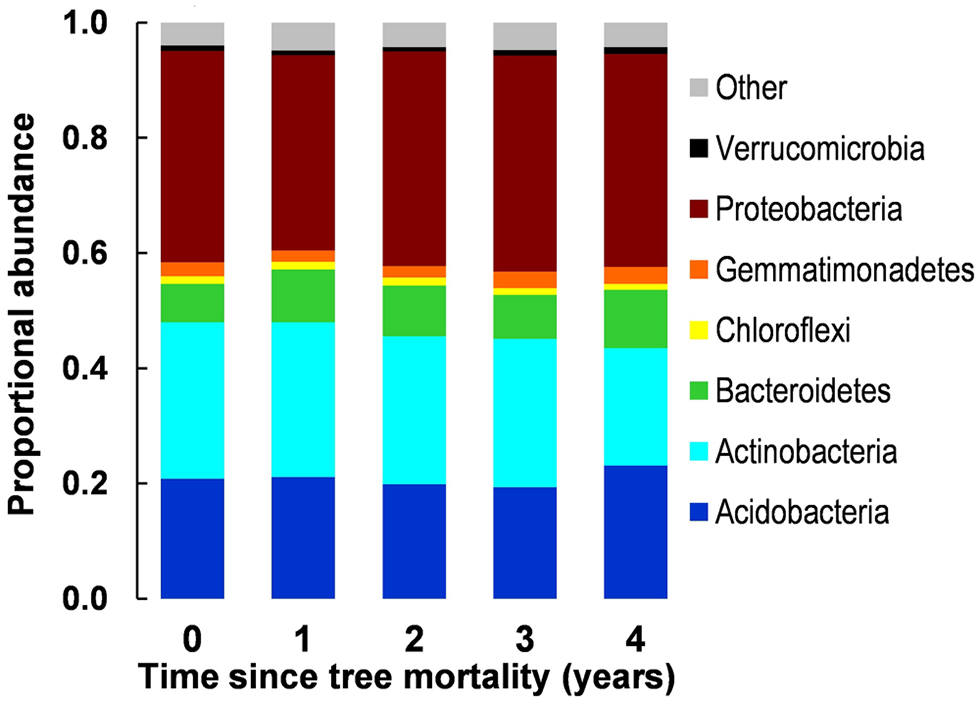
**Proportional abundances of bacterial phyla along a 5-year chronosequence of tree mortality from bark beetle infestations.** Candidate phyla (recently identified phylogentic groups awaiting further description) were grouped together as ‘Other.’

We observed no significant difference in overall community structure among any years of the chronosequence (accessed via MRPP, **Figure 2**) or among proportional abundances of major phyla/subphyla (accessed via χ^2^ tests, **Figure 3**). Tree mortality caused a marginally significant increase in bacterial PD [Kruskal–Wallis χ^2^(4) = 8.22, *P* = 0.084], but did not alter any additional measures of alpha (α) diversity [sample level OTU richness and Shannon diversity (**Table 1**)]. Tree mortality did not significantly influence measures of β-diversity (pairwise dissimilarity of samples) such as pairwise Bray–Curtis and UniFrac measures (**Table 1**).

### COMMUNITY STRUCTURE AND CORRELATIONS WITH ENVIRONMENT

We found relatively few associations (3 out of 35 possible combinations) between bacterial community structure and soil chemistry/environmental variables across the tree mortality chronosequence when examined via Mantel tests. Specifically, we found the bacterial communities from the first year after tree mortality were significantly associated with soil moisture (*r* = 0.58, *P* = 0.024) and pH (*r* = 0.45, *P* = 0.011); while communities from the second year after tree mortality were significantly associated with extractable ammonium (${NH}_{4}^{+}$) concentrations (*r* = 0.54, *P* = 0.006).

**FIGURE 4:**
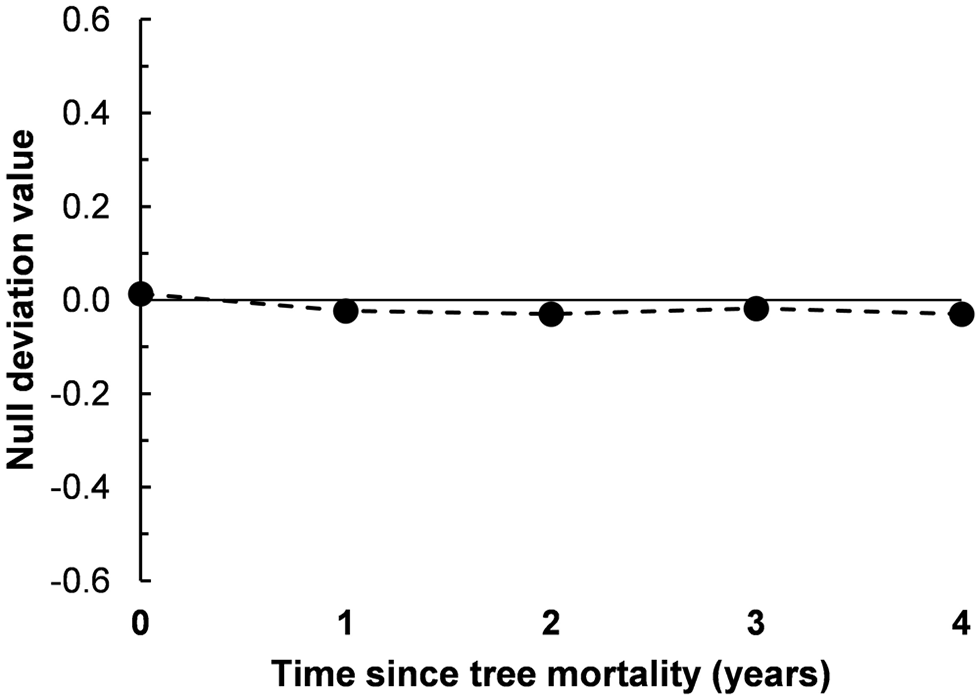
**Null deviation values from soil bacterial communities sampled along a 5-year chronosequence of tree mortality from bark beetle infestations.** Null deviation values close to zero occur when the species (OTU) composition of communities being compared deviates less from random assortments suggesting stochastic processes are important influences structuring the community, larger values (negative or positive) indicate increasing deviation from random and suggest an influence of deterministic processes, possibly due to niche associations.

Using the null deviation approach to assess community assembly processes (Chase and Myers, 2011), we assembled communities *in silico* from the regional species pool to determine if the observed β-diversity patterns deviated from purely stochastic assembly. Null deviation values can range from zero to one, with values closer to zero indicating less deviation from random, suggesting a greater relative influence of stochastic processes. Alternatively, values closer to one (±1) are suggestive of greater deterministic structure, possibly due to niche-associations. We found that null deviation values were very near zero across all 5 years of the bark beetle mortality chronosequence (**Figure 4**), ranging from 0.014 in bacterial communities from soils under live trees (chronosequence year 0) and increasing in absolute value to -0.023, -0.030, -0.018, and -0.030 in years one through four after tree mortality, respectively (**Figure 4**). These increases in null deviation values indicate an increase in deterministic assembly after tree death, but the relatively low values suggest continued influences of stochastic processes on bacterial assemblages over time after tree mortality (**Figure 4**).

### SOIL CHEMISTRY AND N MINERALIZATION/RESIN BAG EXPERIMENTS

**FIGURE 5:**
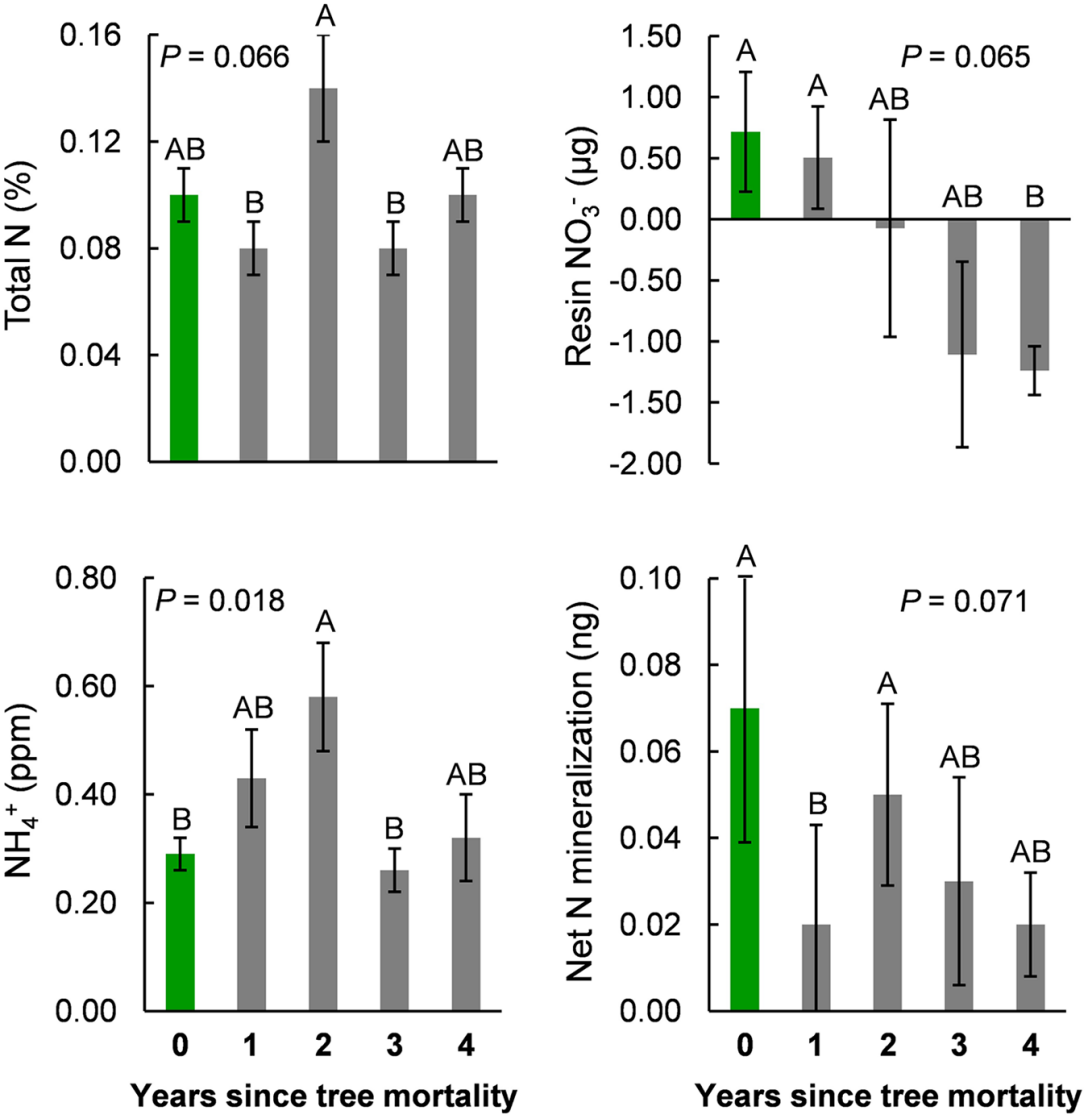
**Measures of total soil N **(upper-left)**, ammonium (${NH}_{4}^{+}$, **lower-left**), plant available nitrogen (${NO}_{3}^{-}$ measured from ion-exchange resin bags, **upper-right**), and net nitrogen mineralization from *in situ* incubation experiments **(lower-right)** across a 5-year chronosequence of bark beetle-induced tree mortality.** Year zero (0, green bars) represents samples collected under live trees that were not attacked by bark beetles during the study. All other samples were collected from under trees attacked and killed by bark beetles one to four (1–4, gray bars) years prior to field sampling.

Tree mortality led to a significant, twofold increase in extractable ${NH}_{4}^{+}$ between living control trees (year 0 of the chronosequence) and year two after bark beetle attack [*F*(4,45) = 3.35, *P* = 0.018; **Figure 5**]. By year three of the chronosequence, ${NH}_{4}^{+}$ had returned to levels similar to those in soils under living control trees (year 0). Total soil N decreased in the first year after tree mortality, but similar to ${NH}_{4}^{+}$, reached its highest levels with a (marginally significant) 40% increase by the second year after tree mortality [*F*(4,45) = 2.37, *P* = 0.066; **Figure 5**]. By the fourth year after tree mortality, total N had returned to a level equivalent to that found in soils under living control trees (**Figure 5**). Total soil C and DOC also increased to peak levels by the second year after tree mortality, but this increase was not significant (**Table 2**) due to high intra-annual variation. Percent soil moisture increased steadily, but not significantly, over time following tree mortality from 4.4% in unaffected soils to 6.4% by the fourth year after trees died (**Table 2**). Similarly, soil pH (mean pH = 6.4 across the chronosequence) was not significantly affected (**Table 2**) by tree mortality.

**Table 2:** Edaphic factors (not including nitrogen) measured at the time of bacterial sampling along a chronosequence of bark beetle-induced tree mortality.

Years since tree mortality^†^	Moisture (%)		DOC (mg/g soil)		Total C (%)		pH	
0	4.4	(±0.5)	0.06	(±0.01)	2.42	(±0.25)	6.3	(±0.1)
1	4.7	(±1.1)	0.08	(±0.02)	2.21	(±0.30)	6.5	(±0.1)
2	5.2	(±0.9)	0.11	(±0.03)	3.24	(±0.56)	6.3	(±0.1)
3	6.4	(±1.1)	0.06	(±0.01)	2.04	(±0.20)	6.6	(±0.1)
4	6.4	(±1.4)	0.07	(±0.01)	2.48	(±0.29)	6.4	(±0.1)

*P-*value	>0.10		0.082		>0.10		>0.10	

*^†^Year 0 = unattacked control trees. *P-*values are from one-way ANOVA on log transformed data; all values are presented as untransformed means ± 1 SE. Soil moisture was determined from gravimetric dry-down methods, DOC, dissolved organic carbon.*

We found a 272% decrease in plant available N (resin bag ${NO}_{3}^{-}$) from control soils under living trees (year 0) to the last year (year 4) of our chronosequence following tree mortality, which, due to high variability was a marginally significant change [Kruskal–Wallis χ^2^(4) = 8.83, *P* = 0.065] (**Figure 5**). Net N mineralization rates declined by 115% in the first year after tree mortality [also marginally significant due to variation within years; Kruskal–Wallis χ^2^(4) = 8.61, *P* = 0.071; **Figure 5**], but returned to levels similar to those found in control soils by the second year after tree mortality.

### N-MINERALIZATION AND BACTERIAL/ENVIRONMENTAL FACTORS

Using stepwise regression we identified best fit models linking bacterial PD, microbial biomass, and soil environmental measures as independent variables and net N mineralization as the dependent variable. The model with the overall best fit (accessed via BIC score) did not retain any environmental measures (**Table 3**), but did retain PD (*P* = 0.47), microbial biomass (*P* = 0.044) and the interaction of PD × microbial biomass (*P* = 0.025) as significantly associated with net N mineralization rates (*R^2^* = 0.31, **Table 3**).

**Table 3:** Best fit model linking soil microbial biomass and bacterial phylogenetic diversity to net nitrogen mineralization across a chronosequence of tree mortality.

*Predictor variables*^†^	*F*	*P*
PD × microbial biomass	5.94	0.025
Microbial biomass (mg/g soil)	3.70	0.044
Phylogenetic diversity (PD)	3.62	0.047

Model *R^2^* = 0.31, *BIC* = *-*39.6		

*^†^Predictor variables were selected via stepwise multiple-regression with smallest BIC (Bayesian information criterion) determing the best model. In addition to microbial variables, starting possible factors included %N, %C, DOC, C:N, pH, soil moisture, and soil temperature.*

## DISCUSSION

We investigated the effects of insect-induced tree mortality on soil bacterial community structure and assembly, and on soil N biogeochemical processes using a 5-year chronosequence of mountain pine beetle (*Dendroctonus ponderosae*) infestations in the southern Rocky Mountains, CO, USA—a region that has experienced an unprecedented level of tree mortality during recent bark beetle epidemics (Meddens et al., 2012). Counter to our hypothesized shifts in bacterial community structure, we found only moderate increases in bacterial phylogenetic diversity (PD, **Table 1**) over time following tree mortality, and no significant changes in microbial biomass, OTU Shannon diversity (H′), pairwise community dissimilarity (i.e., Bray–Curtis dissimilarity and pairwise UniFrac distance), and overall community structure (**Figures 2** and **3**). While we found few associations among soil environmental properties and bacterial community structure, Mantel tests revealed significant relationships among community structure and soil moisture and pH in the first year after tree mortality, and community structure and ammonium (${NH}_{4}^{+}$) in the second year after tree mortality. Null deviation values also indicated that tree mortality led to a minor increase in deterministic influences on community structure, but overall, stochastic processes seemingly remained important for bacterial communities across the chronosequence (**Figure 4**).

Soil properties such as pH, moisture, and C and N concentrations are known to influence soil microbial diversity and activity at both local and global scales (Fierer and Jackson, 2006; Gundersen et al., 2006; Curiel Yuste et al., 2007; Reed and Martiny, 2007; Strickland et al., 2009; Philippot et al., 2013; Landesman et al., 2014). Tree mortality can alter these soil properties as litter inputs and root deposition from mature trees often influence local to regional edaphic factors (Finzi et al., 1998; Chapman et al., 2006; Ushio et al., 2008, 2010; Thoms et al., 2010; Weber and Bardgett, 2011), and both spatial and temporal patterns of microbial diversity have been linked to gradients in tree physiology, litter chemistry, dominant species cover, and canopy tree mortality (Ushio et al., 2008; Thoms et al., 2010; Weber and Bardgett, 2011; Landesman et al., 2014; Štursová et al., 2014). The greater deterministic influences on community assembly in the first year after tree mortality (**Figure 4**), along with the association of bacterial community structure with soil moisture, pH, and ${NH}_{4}^{+}$ in the first and second years after tree mortality coincided with the largest changes in soil chemical properties along the mortality chronosequence (**Table 2**; **Figure 5**). Specifically, tree mortality was followed by a significant increase (*P* < 0.05) in ammonium (${NH}_{4}^{+}$) and a moderate increase (*P* < 0.10) in both total N concentration (%N) and DOC, and a non-significant but notable increase in total C concentration (%C) with all of these measures peaking in the second year after tree mortality.

While tree mortality in other coniferous forest ecosystems has been linked to large changes in soil microbial biomass and community structure (Högberg et al., 2007; Štursová et al., 2014), we found relatively muted effects of tree death on microbial community measures by comparison. A possible explanation is that tree mortality in our study system had little impact on key soil chemical and physical properties known to influence microbial communities in forested ecosystems (Högberg and Högberg, 2002; Monson et al., 2006; Högberg et al., 2007; Weintraub et al., 2007). However, other studies of tree mortality (from girdling experiments) completed < 1 km from our field sites also found moderate effects of tree death on microbial biomass and enzyme activity despite significant changes in soil N concentrations and DOC (Weintraub et al., 2007) and weaker than expected effects on soil processes governed by microbes (Moore et al., 2013). So while it is likely that tree mortality has smaller impacts on soil microbial communities and ecosystem processes in this study system compared to others, the stability of community structure in the face of variable N pools found here and by others suggests a general resistance of soil microbial communities to disturbance of the forest canopy.

Disturbances have long been known to influence species diversity and abundance patterns in macro-biological communities and the majority of microbial communities studied to date have been sensitive to a range of disturbance types, severities, and durations (Allison and Martiny, 2008; Shade et al., 2012). Nevertheless, the structure of bacterial communities in our study resisted bark beetle-induced tree mortality despite concurrent changes in soil N pools and cycling processes (**Table 1**; **Figure 5**). The stability of communities in response to perturbation relies on the ability of individual bacteria to tolerate, endure, or adapt to environmental change. A range of life history strategies facilitate bacterial resistance to disturbance such as: periods of reduced metabolic activity or dormancy, rapid dispersal and colonization, stochastic gene expression, or high efficiency in resource use which is common to oligotrophic bacteria (Lennon and Jones, 2011; Shade et al., 2012; de Vries and Shade, 2013). Communities in our study system are marked by a comparatively high proportions of phyla that contain an abundance of gram-positive taxa (roughly 26% across samples, **Figure 3**) which are often relatively slow growing (a traitcommon to oligotrophs) and also can use dormancy in order to survive disturbance events (Drenovsky et al., 2010; de Vries and Shade, 2013). Thus, the resistance of soil bacterial community structure to environmental changes linked to tree mortality from bark beetle infestations might be explained by the relatively large proportion of oligotrophic taxa found in our study system.

While environmental factors can have important influences on the assembly and structure of microbial communities, a growing body of evidence indicates that bacterial communites can also be strongly influenced by stochastic processes (Burke et al., 2011; Ferrenberg et al., 2013; Nemergut et al., 2013). Null deviation values across our sampling chronosequence suggest that stochastic processes have relatively strong influences on the bacterial communities of our study site, regardless of time since tree death. Even in chronosequence years with the higher null deviations, which would indicate stronger influences of deterministic processes, the values still remained lower than in bacterial communities of soils in a nearby conifer forest (11 km east, 640 m lower in elevation) sampled 2 months prior to our study (see Ferrenberg et al., 2013). While environmental factors would still influence metabolic processes and tolerances in these stochastically assembled bacterial communities (Nemergut et al., 2013), stochastic influences likely lead to heterogeneous distributions of microbial life history traits which may reduce the effects of environmental change on community structure. At the same time, soil processes may remain more stable in the face of environmental changes in cases where microbes and their functional traits are stochastically dispersed and assembled across larger spatial and temporal scales (e.g., Burke et al., 2011). While this scenario has not been thoroughly investigated for soil microbial communities, a strong influence of heterogeneous distributions of vegetation species on the maintenance of ecosystem functioning following disturbances in coniferous forests has been observed (Chapin et al., 2002; Turner, 2010). In particular, vegetation heterogeneity before and after disturbances such as wildfires has been found to not only mediate temporal disturbance effects, but to also help maintain soil nutrients and processes such as N cycling (Turner et al., 2007).

Similar to other insect epidemics, bark beetle-induced tree mortality has been shown to increase concentrations of ammonium (${NH}_{4}^{+}$), nitrate (${NO}_{3}^{-}$), and in some cases, net mineralization rates in soil organic and mineral horizons (Clow et al., 2011; Xiong et al., 2011; Griffin and Turner, 2012; Keville et al., 2013). We found partial support for our third hypothesis that bark beetle-infestation in our study system would lead to increases in soil N pools and N mineralization rates. Specifically, we found increased concentrations of ${NH}_{4}^{+}$, and decreased plant available N (${NO}_{3}^{-}$) and net mineralization rates in mineral soils (**Figure 5**). While our results are lower in magnitude relative to some measures from other studies—possibly associated with low pools of C and N in our soils—there is overall agreement across studies that bark beetle-induced tree mortality moderately affects soil N. Previous studies have also found differences in N dynamics between organic and mineral soil horizons (Keville et al., 2013) and substantial site variation in net N mineralization rates (Griffin and Turner, 2012) suggesting a need to consider experimental context during interpretations. In particular, Schimel and Schaeffer (2012) hypothesized that microbial processes may be more controlled by physical features and structure in mineral soils than in organic layers (Schimel and Schaeffer, 2012), thereby shifting the relationship between structure and function among soil horizons.

Despite concern that extensive tree mortality from bark beetle epidemics would lead to substantial losses of C and N from impacted forests across western North America, reported effects have fallen short of predictions (Clow et al., 2011; Griffin et al., 2011; Griffin and Turner, 2012; Keville et al., 2013; Moore et al., 2013). This contradiction invokes a key role of soil microbial communities in ecosystem responses to tree mortality. Indeed, in the same ecosystem we studied, Moore et al. (2013) found that both photosynthesis and ecosystem respiration declined concurrently after tree death. While our measures of soil bacterial community structure were collected roughly 3 months prior to our N mineralization experiments and therefore must be conservatively interpreted, we found that bacterial PD and total microbial biomass were significantly correlated with N mineralization rates across the tree mortality chronosequence (**Table 3**). This indication that bacterial community structure is important for N mineralization processes in our forested study site agrees with work in other systems (Fraterrigo et al., 2006; Philippot et al., 2013). Nevertheless, the resistance to disturbance from tree mortality of the bacterial community of this subalpine conifer-forest likely reduces the effects of tree mortality on ecosystem processes and could help to explain the relatively weak effects of bark beetle-induced tree mortality on soil N pools. The influence of bacterial phylogenetic diversity on soil N processes also suggests an interesting hypothesis that spatial heterogeneity resulting from stochastic assembly in bacterial communities may play an important role in the stability of spatial and temporal processes rates.

## CONCLUSION

Recent climatic changes have spurred a worldwide increase in insect-induced tree mortality, raising concern over possible effects on biogeochemical cycles (Wardle et al., 2004; Adams et al., 2010; Hicke et al., 2012). Despite being the primary drivers of terrestrial biogeochemical processes, the detailed responses of soil microbial communities to extensive tree mortality during insect epidemics have received limited study (Štursová et al., 2014). We found that soil bacterial communities resisted disturbance from bark beetle-induced tree mortality along a 5-year chronosequence, and that bacterial community assembly was largely influenced by stochastic processes. Results from our characterizations of soil chemistry and nitrogen mineralization agree with reports from similar forest ecosystems (Clow et al., 2011; Griffin et al., 2011; Griffin and Turner, 2012; Keville et al., 2013), as we found only short-lived, moderate effects of bark beetle-induced tree mortality on soil N pools and mineralization rates. The stochastic assembly of bacterial communities in this system likely promotes landscape heterogeneity of microbial functional traits, which coupled with stability of bacterial communities following tree death could help to explain the muted effects of tree mortality on soil N processes across larger spatial scales. Yet given the paucity of reports detailing resistance to disturbance in microbial communities, determining the community characteristics which confer greater resistance to disturbance (Shade et al., 2012) and the bacterial community compositional-traits that influence biogeochemical processes (Schimel and Schaeffer, 2012) will require additional study.

## Conflict of Interest Statement

The authors declare that the research was conducted in the absence of any commercial or financial relationships that could be construed as a potential conflict of interest. The Associate Editor, Jay Lennon, declares that, despite having collaborated with the author, Diana Nemergut, the review process was handled objectively and no conflict of interest exists.
